# Bone Marrow Stromal Cell Regeneration Profile in Treated B-Cell Precursor Acute Lymphoblastic Leukemia Patients: Association with MRD Status and Patient Outcome

**DOI:** 10.3390/cancers14133088

**Published:** 2022-06-23

**Authors:** Elen Oliveira, Elaine S. Costa, Juana Ciudad, Giuseppe Gaipa, Łukasz Sedek, Susana Barrena, Tomasz Szczepanski, Chiara Buracchi, Daniela Silvestri, Patrícia F. R. Siqueira, Fabiana V. Mello, Rafael C. Torres, Leonardo M. R. Oliveira, Isabelle V. C. Fay-Neves, Edwin Sonneveld, Vincent H. J. van der Velden, Esther Mejstrikova, Josep-Maria Ribera, Valentino Conter, Martin Schrappe, Jacques J. M. van Dongen, Marcelo G. P. Land, Alberto Orfao

**Affiliations:** 1 Internal Medicine Postgraduate Program, Faculty of Medicine, Federal University of Rio de Janeiro (UFRJ), Rio de Janeiro 21941-617, Brazil; elenoliveira@ippmg.ufrj.br (E.O.); siqueirapfr@yahoo.com.br (P.F.R.S.); land.marcelo@gmail.com (M.G.P.L.); 2 Cytometry Service, Instituto de Puericultura e Pediatria Martagão Gesteira (IPPMG), Faculty of Medicine, Federal University of Rio de Janeiro (UFRJ), Rio de Janeiro 21941-912, Brazil; fabivmello@gmail.com (F.V.M.); rafaelctorres@hotmail.com (R.C.T.); leleooliveira93@gmail.com (L.M.R.O.); isabellefayneves@gmail.com (I.V.C.F.-N.); 3 Translational and Clinical Research Program, Centro de Investigación del Cáncer and IBMCC (CSIC-University of Salamanca), Cytometry Service, NUCLEUS, Department of Medicine, University of Salamanca (USAL), Institute of Biomedical Research of Salamanca (IBSAL), 37007 Salamanca, Spain; ciudad@usal.es (J.C.); subadelfa@usal.es (S.B.); 4 M. Tettamanti Foundation Research Center, Department of Pediatrics, University of Milano-Bicocca, 20900 Monza, Italy; g.gaipa@asst-monza.it (G.G.); chiara.buracchi@gmail.com (C.B.); 5 Department of Microbiology and Immunology, Medical University of Silesia in Katowice (SUM), 41-808 Zabrze, Poland; lsedek@sum.edu.pl; 6 Department of Pediatric Hematology and Oncology, Medical University of Silesia in Katowice (SUM), 41-800 Zabrze, Poland; szczep57@poczta.onet.pl; 7 Center of Biostatistics for Clinical Epidemiology, Department of Health Science, University of Milano-Bicocca, 20126 Milano, Italy; daniela.silvestri@unimib.it; 8 Princess Maxima Center for Pediatric Oncology, 3584 CS Utrecht, The Netherlands; e.sonneveld-2@prinsesmaximacentrum.nl; 9 Department of Immunology, Erasmus MC, (EMC) University Medical Center Rotterdam, Dr. Molewaterplein 80, 3015 CN Rotterdam, The Netherlands; v.h.j.vandervelden@erasmusmc.nl; 10 Childhood Leukaemia Investigation Prague, Department of Paediatric Haematology and Oncology, Second Faculty of Medicine, Charles University, University Hospital Motol, 15006 Prague, Czech Republic; ester.mejstrikova@lfmotol.cuni.cz; 11 Institut Català d’Oncologia-Hospital Germans Trias I Pujol, Josep Carreras Research Institute Badalona, Universitat Autònoma de Barcelona, 08193 Barcelona, Spain; jribera@iconcologia.net; 12 Pediatric Hematology-Oncology Unit, Department of Pediatrics, University of Milano-Bicocca, MBBM Foundation/ASST Monza, 20900 Monza, Italy; valentino.conter@gmail.com; 13 Department of Pediatric Hematology, University Hospital Schleswig-Holstein, Campus Kiel, 24105 Kiel, Germany; m.schrappe@pediatrics.uni-kiel.de; 14 Department of Immunohematology and Blood Transfusion, Leiden University Medical Center, 2333 ZA Leiden, The Netherlands; j.j.m.van_dongen@lumc.nl

**Keywords:** B-cell precursor acute lymphoblastic leukemia, stromal cells, mesenchymal stem cells, endothelial cells, bone marrow microenvironment, multiparameter flow cytometry, disease free survival, measurable residual disease

## Abstract

**Simple Summary:**

For the last 20 years, measurable residual disease (MRD) has proven to be a strong prognostic factor in B-cell precursor acute lymphoblastic leukemia (BCP-ALL). However, the effects of therapy on the bone marrow (BM) microenvironment and their potential relationship with MRD and patient outcome still remain to be evaluated. Here, we show that mesenchymal stem cells (MSC) and endothelial cells (EC) are constantly present at relatively low frequencies in normal BM and in most follow-up BM samples from treated BCP-ALL patients. Of note, their levels are independent of the MRD status. From the prognostic point of view, an increased percentage of EC among stromal cells (EC plus MSC) at day +78 of therapy was associated with shorter disease free survival (DFS), independently of the MRD status both in childhood and in adult BCP-ALL. Thus, an abnormally high EC/MSC distribution at day +78 of therapy emerges as an adverse prognostic factor, independent of MRD in BCP-ALL.

**Abstract:**

For the last two decades, measurable residual disease (MRD) has become one of the most powerful independent prognostic factors in B-cell precursor acute lymphoblastic leukemia (BCP-ALL). However, the effect of therapy on the bone marrow (BM) microenvironment and its potential relationship with the MRD status and disease free survival (DFS) still remain to be investigated. Here we analyzed the distribution of mesenchymal stem cells (MSC) and endothelial cells (EC) in the BM of treated BCP-ALL patients, and its relationship with the BM MRD status and patient outcome. For this purpose, the BM MRD status and EC/MSC regeneration profile were analyzed by multiparameter flow cytometry (MFC) in 16 control BM (10 children; 6 adults) and 1204 BM samples from 347 children and 100 adult BCP-ALL patients studied at diagnosis (129 children; 100 adults) and follow-up (824 childhood samples; 151 adult samples). Patients were grouped into a discovery cohort (116 pediatric BCP-ALL patients; 338 samples) and two validation cohorts (74 pediatric BCP-ALL, 211 samples; and 74 adult BCP-ALL patients; 134 samples). Stromal cells (i.e., EC and MSC) were detected at relatively low frequencies in all control BM (16/16; 100%) and in most BCP-ALL follow-up samples (874/975; 90%), while they were undetected in BCP-ALL BM at diagnosis. In control BM samples, the overall percentage of EC plus MSC was higher in children than adults (*p* = 0.011), but with a similar EC/MSC ratio in both groups. According to the MRD status similar frequencies of both types of BM stromal cells were detected in BCP-ALL BM studied at different time points during the follow-up. Univariate analysis (including all relevant prognostic factors together with the percentage of stromal cells) performed in the discovery cohort was used to select covariates for a multivariate Cox regression model for predicting patient DFS. Of note, an increased percentage of EC (>32%) within the BCP-ALL BM stromal cell compartment at day +78 of therapy emerged as an independent unfavorable prognostic factor for DFS in childhood BCP-ALL in the discovery cohort—hazard ratio (95% confidence interval) of 2.50 (1–9.66); *p* = 0.05—together with the BM MRD status (*p* = 0.031). Further investigation of the predictive value of the combination of these two variables (%EC within stromal cells and MRD status at day +78) allowed classification of BCP-ALL into three risk groups with median DFS of: 3.9, 3.1 and 1.1 years, respectively (*p* = 0.001). These results were confirmed in two validation cohorts of childhood BCP-ALL (n = 74) (*p* = 0.001) and adult BCP-ALL (n = 40) (*p* = 0.004) treated at different centers. In summary, our findings suggest that an imbalanced EC/MSC ratio in BM at day +78 of therapy is associated with a shorter DFS of BCP-ALL patients, independently of their MRD status. Further prospective studies are needed to better understand the pathogenic mechanisms involved.

## 1. Introduction

In the past 50 years, the outcome of B-cell precursor acute lymphoblastic leukemia (BCP-ALL) has substantially improved, particularly in childhood, due to advances in risk-adapted chemotherapy protocols based on, e.g., the tumor genetic subtype at diagnosis, the presence of measurable residual disease (MRD) after therapy, and improved supportive care, together with the availability of new target therapies [1,2]. Thus, cure rates of up to 90% may be currently reached in childhood BCP-ALL, which decline progressively with age [3,4]. Despite all these advances, a significant fraction of patients that achieve complete remission including also some MRD-negative cases still relapse and their early identification remains a challenge in the management of BCP-ALL [1,4,5].

Accumulated evidences suggest that development, progression, and resistance to therapy of BCP-ALL tumor cells, depends on both the tumor cell features and the tumor microenvironment [6,7,8,9,10,11]. Thus, several studies have demonstrated the presence of shared leukemia-associated genetic alterations on residual hematopoietic cells and bone marrow (BM) stromal cells [12,13,14,15,16] particularly in adult patients, together with a potential role for the BM microenvironment in the pathogenesis of hematological neoplasms [17,18,19]. In this regard, increased angiogenesis and vascularization in BM of both pediatric ALL and ALL-xenografted mice, together with the ability of B-cell lineage lymphoblasts to produce endothelial growth factors such as VEGF, have been previously reported [20,21]. Despite this, the precise relationship between tumor cells, the residual hematopoiesis and the BM microenvironment have not been fully investigated in depth in ALL.

Stromal BM cells are a key component of the BM hematopoietic microenvironment, which comprises a wide variety of different cell types including osteoblasts, osteoclasts, endothelial cells (EC), perivascular reticular cells, and mesenchymal stem cells (MSC), in addition to mast cells and plasma cells [22]. Those cells control the self-renewal, quiescence, chemotaxis, and differentiation of hematopoietic stem cells (HSCs) and other BM hematopoietic cells [23,24,25]. Furthermore, evidences suggest that BM MSC actively participate in the development and response to treatment of leukemia cells, in both acute and chronic leukemia patients [5,26,27]. Similarly, the presence of *KIT*-mutated MSC in BM from indolent systemic mastocytosis patients has been associated with greater rates of disease progression and a poorer outcome [13]. In addition, the presence of genetic alterations typical of leukemia cells of some ALL patients (e.g., *ETV6-RUNX1*, *TCF3-PBX1*, *KMT2A*, and *BCR-ABL*) on in vitro expanded MSC, together with the presence of the BCR-ABL transcript fusion in BM-derived EC from CML patients, point out the close relationship between leukemia cells and other cells in the BM microenvironment [14,15,16]. Despite all of the above, and the fact that the interaction between leukemia cells and the BM microenvironment has been hypothesized to favor leukemia cell growth, clonal evolution, resistance to therapy, MRD persistence, and disease relapse, the potential impact of BM stromal cells in response to therapy and outcome of BCP-ALL patients still remains largely unknown [5,22,28].

Here we investigated the frequency of two specific subtypes of stromal cells (i.e., EC and MSC) in the BM of children and adults diagnosed with BCP-ALL who were studied both at diagnosis and during follow-up. Our main goal was to determine the relative distribution of both cell types in BM of BCP-ALL patients at different points during therapy and the potential relationship with both the BM MRD status and patient outcome.

## 2. Materials and Methods

### 2.1. Patients, Samples, and Controls

A total of 953 BM samples from 347 children diagnosed with BCP-ALL—171 males and 176 females; median age (range) of 4.4 years (y; 0.2–17 y)—and 251 BM samples from 100 adult BCP-ALL patients were studied at diagnosis (n = 229; children = 129 and adults = 100) and at different time points during follow up (n = 975), including: 350 BM samples (309 children and 41 adults) studied at day +15 of therapy; 348 BM samples (282 children and 66 adults) investigated at day +33; and 277 BM samples (233 children and 44 adults) evaluated at day +78 after starting therapy. In parallel, normal/reactive (i.e., control) BM samples from 10 children—6 boys and 4 girls; median age (range) of 10 y (2–18 y)—and 6 adults—4 men and 2 women; median age (range) of 41 y (23–50 y)—were studied as controls. BM samples were obtained and processed within 24 h after collection at 7 different centers: Federal University of Rio de Janeiro (Rio de Janeiro, Brazil), Medical University of Silesia (Zabrze, Poland), University of Salamanca (Salamanca, Spain), Dutch Childhood Oncology Group (The Hague, The Netherlands), Erasmus Medical Center (Rotterdam, The Netherlands), Charles University (Prague, Czech Republic), Fondazione MBBM/S Gerardo Hospital (Monza, Italy). All BM samples were collected in compliance with the guidelines of local ethics and research committees, according to the Declaration of Helsinki; the study was approved by the local ethics committees of the participating centers. All individuals (and/or their guardians in case of children) gave his/her informed consent to participate in the study. Overall, patients were divided into a discovery cohort of 116 BCP-ALL children from Brazil—116 diagnostic BM samples and 338 follow-up BM samples from 62 boys and 54 girls with a median age (range) of 4.4 y (0.2–15 y) including 110 BM samples studied at day +15, 112 BM samples at day +33 and 116 BM samples analyzed at day +78—and two validation cohorts. As validation cohorts, BCP-ALL children treated in Italy (n = 68) and Poland (n = 6)—37 boys and 37 girls with a median age (range) of 4.4 y (1.4–17 y) for which 211 follow-up BM samples were evaluated, including 67 samples studied at day +15, 70 at day +33 and 74 at day +78 together with 74 adult BCP-ALL patients treated in Spain—42 males and 32 females with a median age (range) of 42 y (18–67 y). Follow-up data was available on 134 BM samples (37 samples investigated at day +15, 57 at day +33 and 40 at day +78). All pediatric patients in both the discovery and the validation cohorts were treated with ALL-BFM oriented protocols (INTERFANT 2006, AIEOP-BFM ALL 2009 and AIEOP-BFM ALL 2017), while adult patients were treated with the HR adult ALL PETHEMA2013 protocol for high-risk non-BCR-ABL adult-ALL, as described elsewhere [29].

### 2.2. Immunophenotypic Studies

BCP-ALL samples were stained at diagnosis with the EuroFlow 8-color-fluorescein isothiocyanate (FITC)/phycoerythrin (PE)/PE-cyanin7(PECy7)/peridinin chlorophyll protein-Cy5.5 (PerCP-Cy5.5)/allophycocyanin (APC)/APC-hilite7 (APC-H7)/Pacific Blue (PacB)/Pacific Orange (PacO)-acute leukemia orientation tube (ALOT) plus the EuroFlow BCP-ALL antibody panel [30]: (*i*) cytoplasmic (Cy) MPO/CyCD79a/CD19/CD34/CD7/surface membrane (Sm) CD3/CyCD3/CD45; (*ii*) CD58/CD66c/CD19/CD34/CD10/CD38/CD20/CD45; (*iii*) CyIgM/CD33/CD19/CD34/CD117+ SmIgM/SmIgλ/SmIgκ/CD45; (*iv*) nuclear (Nu) TdT/CD13/CD19/CD34/CD22/CD24/CD9/CD45; (*v*) CD15^+^ CD65/NG2/CD19/CD34/CD123/CD81/CD21/CD45. Staining of cells was performed as previously described in detail, according to the standard operating procedures (SOP) proposed by the EuroFlow Consortium, available at www.euroflow.org (accessed on 15 May 2022) [30]. In turn, follow-up BM samples from BCP-ALL patients obtained at day +15, day +33, and day +78 of therapy were processed according to the EuroFlow bulk-lysis SOP and stained with the EuroFlow 8-color BCP-ALL MRD antibody panel: (*i*) CD81/CD66c+CD123/CD19/CD34/CD10/CD38/CD20/CD45; (*ii*) CD81/CD73+CD304/CD19/CD34/CD10/CD38/CD20/CD45, as previously described in detail [31]. Briefly, normal B-cell precursors were identified as CD19^+^ CD10^+^ and CD38^++^ cells and sub-classified as pro-B (CD45^lo^ CD19^−^ CD81^++^ CD34^+^ CD10^+^ CD20^−^ CD38^++^ CD66c^−^/CD123^−^ CD73/CD304^−^ cells), pre-BI (CD45^lo^ CD19^+^ CD81^++^ CD34^+^ CD10^++^ CD20^−^ CD38^++^ CD66c^−^/CD123^−^ CD73/CD304^−^ cells), pre-BII cells (CD45^lo/+^ CD19^+^ CD34^−^ CD10^+^ CD20^−/+^ CD38^++^ CD66c^−^/CD123^−^ CD73/CD304^−^ cells). In contrast, leukemic B-cell precursors (i.e., blast cells), were all CD19^+^ precursor cells whose immunophenotype deviated from those described above based on aberrantly high or low antigen expression levels and/or the presence of cross-lineage antigen expression and/or asynchronous maturation profiles compared to normal BCP, as previously described in detail [31,32]. In 7% (68/975) of follow-up BM samples, only one of the two BCP-ALL MRD antibodies combinations was tested, due to low BM sample cellularity (e.g., limited BM cellularity in samples collected at day +15). In addition, for detailed characterization of BM MSC and EC, the following 8-color monoclonal antibody combination was further stained in a subset of 5 follow up BCP-ALL BM samples: (*i*) CD81/MSCA1/CD19/CD34/CD90/CD10/CD271/CD45. Stained cells were measured in FACSCanto II flow cytometers-Becton Dickinson Biosciences (BD), San Jose, CA-equipped with the FACS DiVA software (BD). For instrument set up and calibration, the corresponding EuroFlow SOP were used, as described elsewhere [30]. Prior to data analysis, cellular events from BM samples stained with the two BCP-ALL MRD tubes were merged into a single data file. For data analysis, the Infinicyt software (version 2.0, Cytognos SL, Salamanca, Spain) was used and the relative distribution of MSC and EC in BM (i.e., percentage from all BM nucleated cells after excluding cell debris/doublets and blast cells) as well as the relative distribution of MSC and EC within the stromal cells (number of MSC plus EC) were calculated. MRD-negative was defined based on the presence of <20 immunophenotypically aberrant B-cell precursors (i.e., leukemia cells) in BM by multiparameter flow cytometry (MFC), while MRD-positive indicates presence of leukemia cells in the BM above this cut-off.

### 2.3. Statistical Methods

For all numerical variables, mean (standard deviation) or median (interquartile range and range) values were calculated, while, for categorical variables, frequencies were used. In order to establish the statistical significance of differences observed between two or more than two groups, the Mann–Whitney *U* test and the Kruskal–Wallis test were used, respectively. The percentage of stromal cells from all nucleated BM cells, and of MSC and EC within stromal cells, were dichotomized using the Martingale-based residuals analysis, with the following cut-off values: (i) % of BM stromal cells from all BM nucleated cells > 0.21%; (ii) % of MSC from BM stromal cells > 73%; and (iii) % of EC from BM stromal cells >32%, where BM stromal cells (MSC + EC) equals 100%. The same cut-off values were applied for all samples obtained at day +15, day +33 and day +78 after therapy, for the three patient cohorts analyzed. Next, the pre-selected covariates (*p* < 0.20 in the univariate analysis) were included in a multivariate Cox regression model for predicting patient disease-free survival (DFS), defined as the time of relapse from diagnosis to first relapse or the last follow-up visit. DFS curves were plotted according to the Kaplan–Meier method, and the log-rank test was used to determine the statistical significance of differences between DFS curves. Multivariate analysis was performed with the backward stepwise elimination method, in order to obtain the parsimonious predictive model. For all statistical analyses the SPSS software package (version 18.0, IBM Corp Inc., Chicago, IL, USA) was used. *p*-values < 0.05 were considered to be associated with statistical significance.

## 3. Results

### 3.1. Immunophenotypic Identification and Characterization of Stromal Cells in Bone Marrow

**Figure 1:**
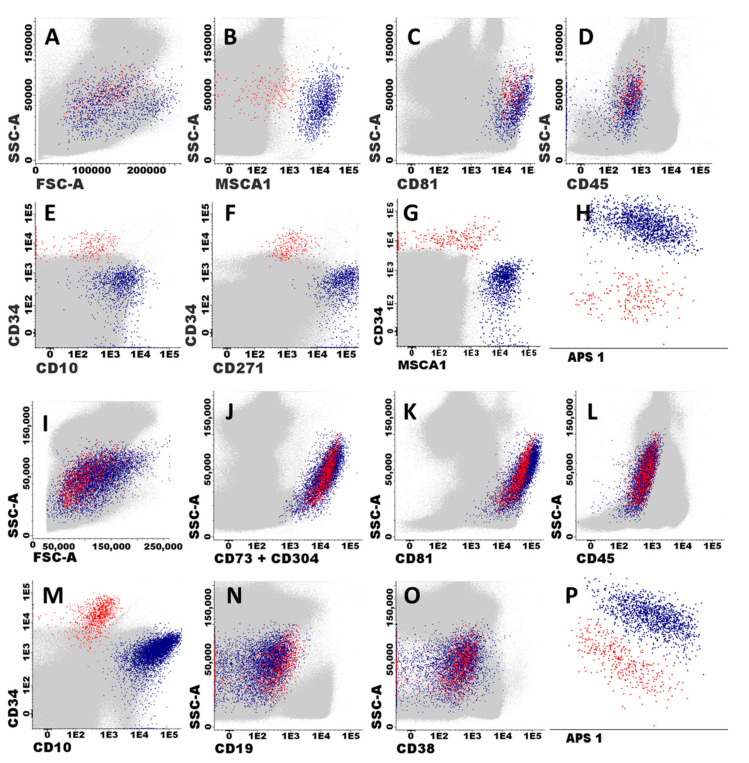
Immunophenotypic identification and characterization of BM stromal cells. Panels (**A**–**H**) illustrate the immunophenotypic pattern of expression of classical stromal cell-associated markers on mesenchymal stem cells (MSC) and endothelial cells (EC). MSC (blue dots) and EC (red dots) shown in all panels were identified as CD81^hi^, (CD73^hi^), and CD45^−^ cells (**B**–**D**), which typically co-expressed MSCA1^+^, CD271^+^, and CD10^hi^ in the absence of CD34 (MSC), or they were CD34^+^ in the absence of MSCA1, CD271, and CD10 expression (EC), respectively. Other BM cells are depicted as grey events. Panels (**I**–**P**) illustrate how BM stromal cells (EC and MSC) were specifically identified and classified after staining with the EuroFlow BCP-ALL MRD antibody panel (Tube 2).

MSC and EC were unequivocally identified in BM based on the following phenotypic criteria: (i) MSC expressed classical MSC-associated markers (MSCA-1^+^, CD73^hi^ and CD271^+^) together with CD81^hi^ and CD10^+^, in the absence of CD34^−^, CD19^−^, and CD45^−^; while (ii) EC were CD81^hi^, CD73^hi^ and CD34^+^, in the absence of CD271^−^, MSCA-1^−^, CD10^−^, CD19^−^, and CD45^−^ (Figure 1). Based on these features, MSC and EC could also be identified with the EuroFlow BCP-ALL MRD antibody panel based on high expression of both CD81 and CD73 in the absence of CD45 and other hematopoietic cell associated markers (CD19, CD66c, CD123, CD38, and CD20) (Figure 1). Among these cells, MSC could be unequivocally discriminated from EC based on their CD10^+^ CD34^−^ phenotype, while EC were positive for CD34 and negative for CD10 (Figure 1). Overall, when we compared the distribution of both stromal cells and their MSC and EC subsets in a subset of BM samples (n = 5), highly similar percent values for the three cell populations were observed once they were identified with the classical MSC and EC vs. the reduced number of markers included in the EuroFlow MRD panel described above: median percentage (range) of 0.035 (0.018–0.054) vs. 0.064 (0.014–0.088) stromal cells (*p* = 0.258), including 26 (10–26) vs. 20 (4–29) EC (p = 0.226) and 75 (74–90) vs. 80 (71–96) MSC (*p* = 0.226), respectively. Based on these highly comparable results, subsequent identification of stromal cells and their MSC and EC subsets was performed in BCP-ALL BM using the markers included in the EuroFlow BCP-ALL MRD panel.

### 3.2. Distribution of MSC and EC in Normal vs. BCP-ALL Bone Marrow

Overall, MSC and EC were both identified in all normal/reactive BM samples investigated (16/16, 100%). Of note, higher percentages of stromal cells (both MSC and EC) were detected in normal/reactive BM from children vs. adults (median of 0.064 vs. 0.014, respectively; *p* = 0.011). Despite this, rather stable and similar percentages of MSC and EC within BM stromal cells, were found in normal/reactive BM from individuals of both age groups (Table 1) Similarly, MSC and EC were also identified in the vast majority of BCP-ALL follow-up samples (874/975, 90%), their frequency increasing from 81% (n = 282/350) of BM samples studied at day +15, to 93% (n = 322/348) and 97% (n = 270/277) of BM samples studied at day +33 and at day +78, respectively. In contrast, stromal cells could not be detected in the BM of children and adults with BCP-ALL studied at diagnosis, except for a small percentage of adults (12%) that showed low numbers of MSC (0.010 ± 0.429%) and EC (<0.001 ± 0.009%) (Table 1).

In the follow up BM samples from BCP-ALL children patients, progressively higher stromal cell percentages were found from day +15 onward, with greater levels in children vs. adults at day +33 (*p* = 0.010) and at day +78 (*p* = 0.025), when normal BM stromal cell levels had been reached (Table 1). Despite adults showing lower stromal cell counts than children, the percentage of BM stromal cells in adults at day +33 (*p* = 0.01) and day +78 (*p* = 0.025) were both abnormally higher than those found in normal/reactive adult BM. Of note, this was also associated with a tendency toward higher EC percentages within BM stromal cells in adult vs. childhood BCP-ALL BM at day +33: median of 27% (0–100) vs. 24% (2–62) (*p* = 0.098) (Table 1).

### 3.3. MRD Status in Follow-Up BM of Childhood and Adult BCP-ALL and Its Relationship with the Distribution of Stromal Cells

**Figure 2:**
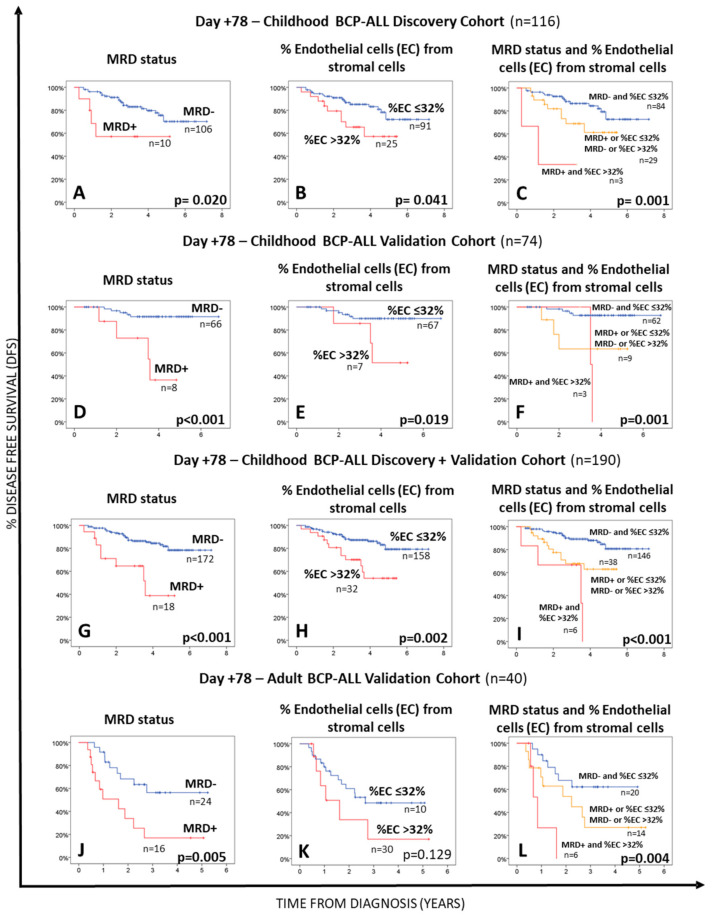
Impact of the BM MRD status Panels (**A**,**D**,**G**,**J**), the percentage of BM endothelial cells Panels (**B**,**E**,**H**,**K**) within the whole BM stromal cell compartment at day +78 or both parameters Panels (**C**,**F**,**I**,**L**) on disease free survival (DFS) of BCP-ALL patients. Panels (**A**–**C**) show data for the discovery patient cohort, while (**D**–**F**,**J**–**L**) panels display survival curves for the validation cohorts of childhood and adult BCP-ALL patients, respectively. DFS curves of all childhood BCP-ALL cases (the discovery plus the validation cohorts) are shown in panels (**G**–**I**). Statistical significance was set at *p* < 0.05 (log rank test).

A similar rate of MRD-positive BM samples was found in children (57%; 470/824 samples) vs. adults (57%; 86/151 BM samples). Once grouped according to the time of follow up, similarly decreasing frequencies of MRD+ BM samples were found at day +15 vs. day +33 in children—281/309 (91%) and 164/282 (58%) (*p* < 0.0001)—compared to adult BCP-ALL-37/41 (90%) at day +15 and 33/66 (50%) at day +33 (*p* < 0.0001), respectively-. In contrast, at day +78 the frequency of MRD+ BM samples was significantly lower (*p* < 0.0001) in children—25/233 (11%)—vs. adults—16/44 (36%). As expected, the presence of MRD in BM at both day +33 and day +78 of therapy was associated with a poorer patient outcome in terms of DFS in the discovery (*p* = 0.033 and *p* = 0.020, respectively) as well as the validation BCP-ALL patient cohorts (*p* = 0.159 and *p* < 0.001 for children and *p* = 0.003 and *p* = 0.005 for adults, respectively) (Table 2, Figure 2A,D,J and Figure S1A,B,D). Of note, similar frequencies of both MSC and EC were detected in the BM of MRD+ vs. MRD- BCP-ALL patients studied at different time points during follow-up (Supplementary Table S1).

### 3.4. Relationship between the Distribution of MSC and EC in BM at Day +78 and Both the MRD Status and Outcome of BCP-ALL Patients

Interestingly, increased percentages of EC within the stromal cell compartment in BM of BCP-ALL patients studied at day +78 above 32% were associated with a significantly shorter DFS in children with BCP-ALL included in the discovery cohort (*p* = 0.041) (Figure 2B, Table 2). Multivariate analysis including all relevant prognostic factors (i.e., age at diagnosis, genetic markers, and the MRD status) together with the percentage of stromal cells at day +33 and the number of EC within BM stromal cells at both day +33 and day +78, showed that the percentage of EC from BM stromal cells, together with MRD (both studied at day +78), was the best combination of independent prognostic factors for DFS in our discovery cohort of childhood BCP-ALL (Table 2 and Supplementary Table S2). Based on these results, we divided our patients into three groups, depending on the MRD status and percentage of EC in BM at day +78. These included: (i) a favorable risk group, consisting of patients who were MRD-negative and had ≤32% of EC in BM at day +78; (ii) an intermediate risk group, which included patients classified as either MRD-positive with ≤32% EC from all BM stromal cells or as MRD-negative with higher numbers (>32%) of EC in BM at day +78, who showed a very similar (intermediate) outcome (5-year DFS probability of 62.9% vs. 64.1%, *p* = 0.514) (Supplementary Figure S2) and (iii) an adverse risk group formed by patients classified as MRD+, which also had increased counts (>32%) of EC in BM at day +78 (Table 2 and Figure 2C). Such prognostic classification was associated with progressively lower median DFS from low-risk to intermediate-risk-HR: 2.12 (95%CI: 0.92–4.86); *p* = 0.001 and high risk-HR: 9.63 (95%CI: 2.14–43.32) patients, with progressively lower median DFS rates of 3.9, 3.1, and 1.1 years, respectively (Table 2). Similar results were observed for the childhood and adult BCP-ALL validation cohorts, with progressively shorter DFS rates for low, intermediate, and high-risk childhood (*p* = 0.001) and adult (*p* = 0.004) (BCP-ALL) patients (Table 3 and Figure 2). Interestingly, MRD^−^ childhood BCP-ALL patients (discovery plus validation cohorts) who presented with >32% EC within BM stromal cells at day +78 had a significant shortened median DFS than those patients that showed lower EC numbers (≤32% EC among all BM stromal cells) with median 5-year DFS rates of 64.1% vs. 80.7%, respectively (*p* = 0.02) (Supplementary Figure S2). Of note, in both validation cohorts the combination of these two parameters (MRD status and the percentage of EC from stromal cells in BM at day +78) also emerged as the most powerful independent prognostic factor for DFS (Table 3).

## 4. Discussion

BCP-ALL consists of a malignant expansion of B-cell precursors blocked at relatively early stages of maturation, caused by various genetic drivers and altered genomic profiles [33]. For decades, major efforts have been focused on detailed characterization of the genetic events that affect blast cells in BCP-ALL for better understanding of the ontogeny and heterogeneous clinical behavior of this group of diseases, and identifying more efficient therapies [34]. Despite this, an increased number of evidences point out the potential existence of a multifactorial interplay between an underlying genetic predisposition and an altered interaction between the tumor cell, normal residual hematopoiesis, and the BM microenvironment, favoring the development of BCP-ALL and its clonal evolution and expansion as well as resistance to therapy, persistence of residual disease, and eventually also disease relapse [5,11,28]. In this study, we investigated for the first time the BM stromal cell regeneration profile and the relative distribution of MSC and EC in BM of a large cohort of children and adults with BCP-ALL studied both at diagnosis and during treatment, compared with normal/reactive BM. Our ultimate goal was to determine the potential relationship between the BM stromal cell regeneration profile and both the MRD status and patient outcome.

Several studies have previously reported that hematopoietic niches become sanctuaries where leukemia cells can be protected from the effects of chemotherapy and, thereby, acquire a resistant phenotype [35,36,37,38]. For instance, deletion of the IKZF1 gene in BCR-ABL1-positive BCP-ALL patients has been shown to not confer in vitro resistance to chemotherapy on its own, although resistance might occur due to new interactions between the leukemia cells presenting IKZF1 deletion and their BM microenvironment [38]. These findings are in line with other observations that indicate that leukemia cells might even be able to modify and remodel the BM niches to support their survival and proliferation [35,36,37].

Several research groups have investigated and characterized the BM stroma of patients with a broad variety of distinct hematological malignancies [5,14,15,23,26,39]. Despite this, in most of those studies, in vitro expansion of stromal cells was used prior to their characterization, which may modify their immunophenotypic, functional and/or even, genetic characteristics, leading to conflicting results in the literature regarding the altered features of, e.g., BM MSC in BCP-ALL and other hematological malignancies [40,41,42]. Only a few studies have so far characterized the ex vivo immunophenotype of normal BM MSC and EC, but in diseases other than BCP-ALL [43,44,45].

Recently, the EuroFlow consortium developed a standardized and high sensitive next-generation flow cytometry (NGF) assay for MRD monitoring in BCP-ALL [31]. The high number of BM cells evaluated and the specific combination of markers (CD45, CD81, CD73, CD34 and CD10) [45,46,47,48,49] allowed accurate ex vivo identification/quantification of minor populations of BM cells such as MSC and EC, simultaneously with MRD. In order to verify if such markers can be routinely used to specifically identify MSC and EC, we tested them in combination with other stromal cells-associated markers, such as CD271, CD90, and MSCA-1, in addition to CD81, CD10, CD34, CD19, and CD45 [45]. Once we stained BM samples with these markers in parallel to the EuroFlow BCP-ALL MRD panel, we could clearly demonstrate that BM stromal cells can be unequivocally detected with the later panel, based on high expression of CD73 and CD81 in the absence of CD45 and other hematopoietic markers (i.e., CD19, CD66c, CD123, CD38, and CD20). Within these BM stromal cells, the two minor populations of CD10^+^ CD34^−^ MSC and CD10^−^ CD34^+^ EC, could also be further accurately discriminated, as previously described [45,46,47,48,49,50,51].

Further analysis of the distribution of both populations of MSC and EC in BM showed that they are systematically present at low frequencies in both childhood and adult normal/reactive BM, in line with previous observations of MSC in normal BM and EC in BM from patients with, i.e., alcoholic liver cirrhosis and myelodysplastic syndromes [45,52,53,54]. In our study, significantly lower numbers of BM stromal cells were detected in adult vs. childhood normal/reactive BM samples, which might reflect the impact of aging in the BM stroma in parallel to the changes in hematopoiesis [3,4]. Despite this, a similar proportion of MSC and EC was observed within the BM stromal cell compartment in children vs. adults. In contrast, BM samples from BCP-ALL patients studied at diagnosis were virtually depleted of stromal MSC and EC, except for a minor subset of adult BCP-ALL patients. However, this may be due to the relatively lower numbers of cells analyzed at diagnosis in the context of massive infiltration by leukemia cells, pointing out the need for further prospective studies with millions of BM cells also studied at diagnosis.

In contrast to diagnostic BM, we identified the presence of stromal cells in the vast majority of BCP-ALL evaluated during treatment, which progressively increased over normal values from day +15 to day +33 and day +78. Of note, at day +15 of therapy, abnormally low or undetected stromal cell counts vs. normal/reactive BM, were found in children with BCP-ALL, which is most likely due to the chemotherapy-induced BM aplasia, expected at the early phases of treatment, and the potential effect of hemodilution on the distribution of these cells [55,56]. Conversely, increased numbers of total stromal cells were observed in BM from children at day +33 after therapy, reaching normal values at day +78 after therapy. These results are consistent with previous in vitro studies concerning MSC in childhood BCP-ALL BM, in which chemotherapy did not seem to the hamper isolation of these cells during treatment [39]. In contrast, BM stromal cells from adult BCP-ALL BM appear to be systematically not affected at the very early phases of chemotherapy, with persistently increased percentages of stromal cells (vs. normal/reactive adult BM) throughout treatment. These results may reinforce the potential involvement of the microenvironment in the maintenance of leukemia BM niches in adult BCP-ALL outcome vs. childhood patients [35,36,37,38,57]. Based on this hypothesis, a close association would be expected between the stromal cells counts and the BM MRD status. However, no significant differences were found between the distribution of stromal MSC and EC according to the BM MRD status in childhood vs. adult BCP-ALL.

In order to gain further insight into the potential role of BM stromal cells on the outcome of BCP-ALL, we investigated the prognostic impact of the distribution of such cells in childhood BCP-ALL at different time points after starting therapy, compared to other well-established prognostic factors such as MRD [32,58,59,60,61,62,63,64,65,66,67,68,69] and tumor cytogenetics [70,71,72,73,74]. Interestingly, our results showed that increased percentages of EC within stromal cells found at day +78 of therapy identified a poorer prognosis subgroup of childhood BCP-ALL, independently of the BM MRD status. A combined assessment of the MRD status and the percentage of EC within stromal cells at day +78 proved to be the most powerful combination of independent prognostic factors for predicting DFS in the discovery and validation cohorts of childhood and adult BCP-ALL patients analyzed. In the last decades, multiple studies have systematically shown the prognostic value of the BM MRD status in both childhood and adult BCP-ALL, independently of the therapeutic protocols used [65,66,67,68,69,75,76]. This has led to the progressive incorporation of the BM MRD status on prospective clinical trials and other treatment protocols with a significant benefit to patient survival. Among other, day +78 MRD has emerged as one of the most informative time points for predicting patient outcome. In contrast, the role of the BM microenvironment, particularly of stromal cells such as MSC and EC on determining response to therapy and outcome of BCP-ALL patients remains to be investigated.

To the best of our knowledge, this is the first time in which the increase in EC (with a decrease in MSC) within the BM stroma of treated BCP-ALL patients is associated with a poorer prognosis. Despite the specific mechanisms involved deserving further investigations, it might be hypothesized that such an unfavorable prognostic impact could be due to the potential role of EC in supporting leukemia cell survival and proliferation, besides protecting them from chemotherapy-induced apoptosis [77,78] and/or the down-regulated immunomodulatory and anti-tumoral role of the decreased MSC compartment [79,80]. Thus, previous studies have shown that activation of EC promote their adhesion to leukemia cells, a condition that also confers both a proliferative advantage and a decreasing susceptibility to chemotherapy to the leukemia cells [78]. In turn, the increased numbers of EC in BM may also reveal an underlying increased neoangiogenesis in these patients [14], similarly to what has been reported in acute myeloblastic leukemia (AML), where EC appears to trigger (or be associated with) inflammatory responses related with the activation of leukemia cell and disease progression [81]. In line with these findings, more recent studies suggested that specific *IKZF1* gene alterations may also induce an increased adhesion of BCP-ALL BM blast cells to their microenvironment, which would contribute to the activation of cell signaling pathways that contribute to leukemia cell resistance against chemotherapy [37]. Whether the *IKZF1* alteration status is associated or not with increased EC cell percentages (and also decreased MSC) among stromal cells in BCP-ALL still remains to be elucidated.

## 5. Conclusions

In this study, we describe for the first time the treatment-associated BM regeneration profile of stromal cells (MSC and EC) in children vs. adult BCP-ALL patients and their relationship with the BM MRD status and patient disease free-survival. Overall, our results show that the combined assessment of the BM MRD status and the percentage of EC within BM stromal cells at day +78 of therapy emerges as a novel powerful combination of prognostic factors for risk stratification of both children and adult BCP-ALL patients treated with (different) currently used chemotherapy-based regimens. Further investigations in larger independent series of childhood and adult BCP-ALL patients are required to better understand the mechanisms involved in determining the association reported here between the composition of the BM stromal cell compartment during therapy and the outcome of BCP-ALL patients.

## Figures and Tables

**Table 1:** Distribution of stromal cells in normal/reactive bone marrow (BM) and both diagnostic and follow-up BM samples from children vs. adult patients diagnosed with BCP-ALL.

Samples	% BM Stromal Cells-From All BM Cells	% Mesenchymal Cells-From BM Stromal Cells	% Endothelial Cells-From BM Stromal Cells	% Blast Cells by FCM
**Normal/Reactive BM Donors**				
Children (n = 10)	0.064 (0.014–0.087)	80 (71–96)	20 (4–29)	-
Adults (n = 6)	0.014 (0.005–0.037)	81 (75–90)	20(10–25)	-
***p*-value**	**0.011**	0.875	0.875	-
**Diagnostic BCP-ALL BM**				
Children (n = 129 ^∆^, n = 0 ^♥^)	0	0	0	84 (20–99)
Adults (n = 100 ^∆^, n = 12 ^♥^)	0 (0–0.44)	100 (25–100)	0 (0–75)	81 (30–97)
***p*-value**	**<0.001**	NA	NA	0.293
** Day +15 BCP-ALL BM **				
Children (n = 309 ^∆^, n = 247 ^♥^)	0.029 (0–1.57)	76 (13–99)	24 (1–87)	0.45 (0–84)
Adults (n = 41 ^∆^, n = 35 ^♥^)	0.04 (0–0.95)	76 (15–100)	24 (0–85)	1.1 (0–91)
***p*-value**	0.541	0.802	0.802	0.087
**Day +33 BCP-ALL BM**				
Children (n = 282 ^∆^, n = 266 ^♥^)	0.048 (0–1.85)	76 (38–98)	24 (2–62)	0.002 (0–56)
Adults (n = 66 ^∆^, n = 57 ^♥^)	0.029 (0–1.11)	73 (0–100)	27 (0–100)	0.001 (0.001–77)
***p*-value**	**0.010**	0.097	0.098	0.705
**Day +78 BCP-ALL BM**				
Children (n = 233 ^∆^, n = 228 ^♥^)	0.063 (0–1.22)	76 (40–95)	24 (5–60)	0 (0–11)
Adults (n = 44 ^∆^, n = 42 ^♥^)	0.043 (0–0.063)	76 (38–98)	24 (2–62)	0 (0–63)
***p*-value**	**0.025**	0.863	0.867	**<0.001**

Abbreviations: BM: bone marrow; BCP-ALL: B-cell precursor acute lymphoblastic leukemia; NA: not applied; ^∆^ Total number of samples analyzed; ^♥^ Number of samples in which stromal cells were present.

**Table 2:** Univariate and multivariate analysis of prognostic factors for disease-free survival (DFS) of children diagnosed with BCP-ALL included in the discovery cohort (n = 116).

	Univariate Analysis				Multivariate Analysis			Multivariate Analysis *		
	Median DFS (Years)	HR	95th CI	*p*-Value	HR	95th CI	*p*-Value	HR	95th CI	*p*-Value
**CHILDHOOD BCP-ALL**										
**Discovery Cohort**										
**Age at diagnosis**										
≥1 to <10 years	5.60	1								
<1 or ≥10 years	4.67	2.62	(1.14–6.03)	**0.024**						
**Genetic Abnormalities**										
Favorable	5.95	1								
Adverse	3.01	3.30	(1.02–10.65)	**0.046**						
**MRD Status**										
**Day +33** MRD^−^	5.88	1								
MRD^+^	5.40	2.62	(1.04–6.60)	**0.041**						
**Day +78** MRD^−^	5.92	1			1					
MRD^+^	3.30	3.32	(1.13–9.73)	**0.029**	3.28	(1.12–9.66)	**0.031**			
**% Stromal cells**										
**Day +33** ≤0.21%	6.02	1								
>0.21%	3.85	3.04	(1.20–7.70)	**0.019**						
**% Endothelial cells (EC)**										
**Day +78** ≤32%	6.01	1			1					
>32%	3.97	2.27	(1.01–5.10)	**0.048**	2.50	(1–9.66)	**0.05**			
**MRD Status and %EC**										
**Day +78**										
MRD^−^ and %EC ≤ 32%	6.09	1		**0.001**				1		**0.001**
MRD^+^ or %EC ≤ 32% MRD^−^ or %EC > 32%	4.13	2.12	(0.92–4.86)					2.12	(0.92–4.86)	
MRD^+^ and %EC > 32%	1.56	9.63	(2.14–43.32)					9.63	(2.14–43.32)	

* Multivariate analysis including the contribution of MRD status and %EC at day +78. Abbreviations: CI: confidence interval; HR: hazard ratio; MRD: minimal residual disease; EC: endothelial cells.

**Table 3:** Prognostic impact of the new BCP-ALL risk stratification model proposed for DFS of patients included in the childhood BCP-ALL validation cohort, the childhood BCP-ALL discovery + validation cohorts, and the adult BCP-ALL validation cohort.

	Univariate Analysis				Multivariate Analysis		
	Median DFS (Years)	HR	95th CI	*p*-Value	HR	95th CI	*p*-Value
**CHILDHOOD BCP-ALL**							
**Validation Cohort**							
**MRD Status** **Day +78** MRD^−^	6.43	1					
MRD^+^	3.49	8.25	(2.20–30.84)	**0.002**			
**%Endothelial cells (EC)** **Day +78** ≤32%	6.35	1					
>32%	4.16	4.52	(1.13–18.10)	**0.033**			
**Day +78 MRD Status and % EC**							
MRD^−^ and %EC ≤ 32%	6.50	1		**0.001**	1		**0.001**
MRD^+^ or %EC ≤ 32% MRD^−^or %EC > 32%	3.94	6.35	(1.42–28.50)		6.35	(1.42–28.50)	
MRD^+^ and %EC > 32%	3.54	9.81	(1.79–53.88)		9.81	(1.79–53.88)	
**Discovery plus Validation Cohort**							
**MRD Status** **Day +33** MRD^−^	6.30	1					
MRD^+^	5.74	2.64	(1.14–6.08)	**0.023**			
**Day +78** MRD^−^	6.24	1					
MRD^+^	3.30	4.34	(1.96–9.63)	**<0.001**			
**% Stromal cell at** **Day +33** ≤0.21%	6.25	1					
>0.21%	4.55	3.17	(1.47–6.85)	**0.003**			
**% Endothelial cells (EC) Day +78** ≤32%	6.28	1					
>32%	4.00	2.88	(1.43–5.80)	**0.003**			
**Day +78 MRD Status and % EC**							
MRD^−^ and %EC ≤ 32%	6.38	1			1		
MRD^+^ or %EC ≤ 32% MRD^−^ or %EC > 32%	4.13	2.92	(1.41–6.03)	**<0.001**	2.92	(1.41–6.03)	**<0.001**
MRD^+^ and %EC > 32%	2.60	8.03	(2.69–24.02)		8.03	(2.69–24.02)	
**ADULT BCP-ALL**							
**Validation Cohort**							
**MRD Status** **Day +33** MRD^−^	3.87	1					
MRD^+^	1.80	3.40	(1.41–8.18)	**0.006**			
**Day +78** MRD^−^	3.64	1					
MRD^+^	1.94	3.26	(1.28–8.32)	**0.014**			
**Day +78 MRD Status and % EC**							
MRD^−^ and %EC ≤ 32%	3.64	1		**0.004**	1		**0.004**
MRD^+^ or %EC ≤ 32% MRD^−^ or %EC > 32%	2.52	2.99	(0.80–6.63)		2.99	(0.80–6.63)	
MRD^+^ and %EC > 32%	0.92	8.33	(2.08–33.40)		8.33	(2.08–33.40)	

Abbreviations: CI: confidence interval; HR: hazard ratio; MRD: minimal residual disease; EC: endothelial cell.

## Data Availability

The data presented in this study are available on request from the corresponding author.

## Supplementary Materials

The following supporting information can be downloaded at: https://www.mdpi.com/article/10.3390/cancers14133088/s1, Table S1: Distribution of MSC and EC in normal and both childhood and adult BCP-ALL bone marrow (BM) according to the BM MRD status at day +15, day +33 and day +78 after starting therapy; Table S2: Univariate and multivariate analysis of prognostic factors for disease-free survival (DFS) of patients included in the discovery cohort of childhood BCP-ALL (n = 116); Figure S1: Impact of the BM MRD status and the percentage of BM endothelial cells within the whole BM stromal cell compartment (Panels A–C) at day +15 and day +33 of therapy on disease free survival (DFS) of childhood BCP-ALL (panel A: discovery cohort; B: validation cohort; C: discovery plus validation cohort) and adult BCP-ALL (Panel D), respectively. Statistical significance was set at *p* < 0.05 (log-rank test); Figure S2: Impact of the bone marrow (BM) minimal residual disease (MRD) status and the percentage of BM endothelial cells (EC) within the whole BM stromal cell compartment at day +78 of therapy on disease-free survival (DFS) of childhood BCP-ALL for both the discovery plus validation cohorts: comparison between MRD^−^ patients with >32% EC in BM vs. MRD^+^ cases with ≤32% EC (Panel A), and among MRD^−^ patients between cases with low (≤32%) vs high (>32%) percentages of EC within BM stromal cells (Panel B). Statistical significance was set at *p* < 0.05 (log-rank test). * Breslow test was used in Panel A to exclude a possible statically significant difference at the first part of the curve.
