# Supplementary material for: Bone Marrow Stromal Cell Regeneration Profile in Treated B-Cell Precursor Acute Lymphoblastic Leukemia Patients: Association with MRD Status and Patient Outcome
Source: Cancers (Basel). 2022 Jun 23;14(13):3088. doi: 10.3390/cancers14133088 (PMC9265080; doi:10.3390/cancers14133088)
Supplement: Supplementary file 1 [file cancers-14-03088-s001.zip › cancers-1751219-supplementary.pdf]

Table S1. Distribution of MSC and EC in normal and both childhood and adult BCP-ALL bone marrow (BM) according to the BM MRD status at day +15, day +33, and day +78 after starting therapy.

Samples		% BM Stromal Cells From BM Cells	% Mesenchymal Cells From BM Stromal Cells	% Endothelial Cells From BM Stromal Cells	% Blast Cells
**Normal/Reactive Donors**					
Children (n = 10)		0.064 (0.014–0.087)	80 (71–96)	20 (4–29)	-
Adults (n = 6)		0.014 (0.005–0.037)	81 (75–90)	20 (10–25)	-
***p*- value ***		**0.011**	0.875	0.875	-
**BCP-ALL**					
**Day +15 after therapy**					
Children	MRD^{+} (n = 281 △, n = 226 ♥)	0.028 (0–1.57)	77 (13–99)	23 (1–87)	0.620 (0.0002–84)
	MRD^{-} (n = 28 △, n = 21♥)	0.032 (0–0.40)	74 (30–93)	26 (7–70)	0
***p*-value ***		0.929	0.437	0.436	**<0.001**
Adults	MRD^{+} (n= 37△, n = 32♥)	0.030 (0–0.95)	73 (15–100)	27 (0–85)	2.000 (0.01–91)
	MRD^{-} (n = 4 △, n = 3 ♥)	0.330 (0–0.57)	94 (77–96)	6 (4–23)	0
***p*-value ***		0.146	0.125	0.125	**<0.001**
**Day +33 after therapy**					
Children	MRD^{+} (n= 164 △, n = 158 ♥)	0.051 (0–1.85)	76 (49–98)	24 (2–51)	0.018 (0.0002–56)
	MRD^{-} (n = 118 △, n = 108 ♥)	0.045 (0–1.52)	76 (38–97)	24 (3–62)	0
***p*-value ***		0.417	0.226	0.229	**<0.001**
Adults	MRD^{+} (n = 33 △, n = 29 ♥)	0.039 (0–0.3)	67 (0–94)	33 (6–100)	0.050 (0.002–69)
	MRD^{-} (n = 33 △, n = 28 ♥)	0.020 (0–1.11)	76 (28–100)	24 (0–72)	0
***p*-value ***		0.546	0.076	0.076	**<0.001**
**Day +78 after therapy**					
Children	MRD^{+} (n = 25 △, n= 25 ♥)	0.120 (0.005–0.83)	76 (40–95)	24 (5–60)	0.021 (0.0006–11)
	MRD^{-} (n = 208△, n = 203 ♥)	0.061 (0–1.22)	76 (51–94)	24 (6–49)	0
p*- value		0.351	0.110	0.110	**<0.001**
Adults	MRD+ (n = 16 △, n = 15 ♥)	0.024 (0–0.63)	76 (38–98)	24 (2–62)	0.054 (0.0015–63)
	MRD^{-} (n = 28 △, n = 27 ♥)	0.050 (0–0.4)	76 (48–96)	24 (4–52)	0
***p*-value ***		0.583	0.733	0.733	**<0.001**

Abbreviations: BM: bone marrow; BCP-ALL: B-cell precursor acute lymphoblastic leukemia; ND: not detected; △ Total number of samples analyzed; ♥ Number of samples in which stromal cells were present; * Kruskal–Wallis test.

Table S2. Univariate and multivariate analysis of prognostic factors for disease-free survival (DFS) of patients included in the discovery cohort of childhood BCP-ALL (n = 116).

	Univariate Analysis				Multivariate Analysis		
	**Median DFS (Years)**	**HR**	**95th CI**	***p*-Value**	**HR**	**95th CI**	***p*-Value**
**Age at diagnosis**							
≥ 1 to <10 years	5.60	1					
<1 or ≥ 10 years	4.67	2.62	(1.14–6.03)	**0.024**			
**Gender**							
Female	5.27	1					
Male	5.63	1.34	(0.64–3.22)	0.383			
**WBC at diagnosis**							
≤ 50 × 10^9/L	5.88	1					
>50 × 10^9/L	4.69	1.45	(0.54–3.87)	0.461			
**PB Blasts at Day +8**							
< 1 × 10^9/L	5.95	1					
≥1 × 10^9/L	4.45	1.40	(0.90–2.16)	0.134			
**Genetic Abnormalities**							
Favorable	5.95	1					
Adverse	3.01	3.30	(1.02–10.65)	**0.046**			
**Follow-up MRD:**							
**Day +15**							
MRD$^-$	ND *	1					
MRD$^+$	5.76	23.21	(0.04–13.355)	0.332			
**Day +33**							
MRD$^-$	5.88	1					
MRD$^+$	5.40	2.62	(1.04–6.60)	**0.041**			
**Day +78**							
MRD$^-$	5.92	1			1		
MRD$^+$	3.30	3.32	(1.13–9.73)	**0.029**	3.28	(1.12–9.66)	**0.031**
**% Stromal cells:**							
**Day +15**							
≤ 0.21%	5.78	1					
>0.21%	ND *	0.44	(0–51.9)	0.387			
**Day +33**							
≤ 0.21%	6.02	1					
>0.21%	3.85	3.04	(1.20–7.70)	**0.019**			
	5.92	1					

**Day +78**							
≤ 0.21%							
>0.21%	4.52	1.96	(0.79–4.90)	0.150			
**% Mesenchymal cells:**							
**Day +15**							
≤ 73%	5.53	1					
>73%	5.71	0.49	(0.21–1.17)	1.060			
**Day +33**	4.81	1					
≤ 73%							
>73%	6.13	0.56	(0.25–1.25)	0.555			
**Day +78**	4.99	1					
≤ 73%							
>73%	6.06	0.55	(0.26–1.20)	0.134			
**% Endothelial cells (EC):**							
**Day +15**							
EC ≤ 32%	5.53	1					
EC > 32%	5.44	1.71	(0.72–4.06)	0.226			
**Day +33**	5.93	1					
EC ≤ 32%							
EC > 32%	5.00	1.27	(0.53–3.07)	0.593			
**Day +78**	6.01	1			1		
EC ≤ 32%							
EC > 32%	3.97	2.27	(1.01–5.10)	**0.048**	2.50	(1–9.66)	**0.05**
**MRD Status and % EC at Day +78:**							
MRD^{-} and EC ≤ 32%	6.09	1					
MRD^{+} or EC ≤ 32%<br>MRD^{-} or EC > 32%	4.13	2.12	(0.92–4.86)	**0.001**			
MRD^{+} and EC > 32%	1.56	9.63	(2.14–43.32)				

CI: confidence interval; HR: hazard ratio; ND: not determined; MRD: minimal residual disease * there were no events in this group.

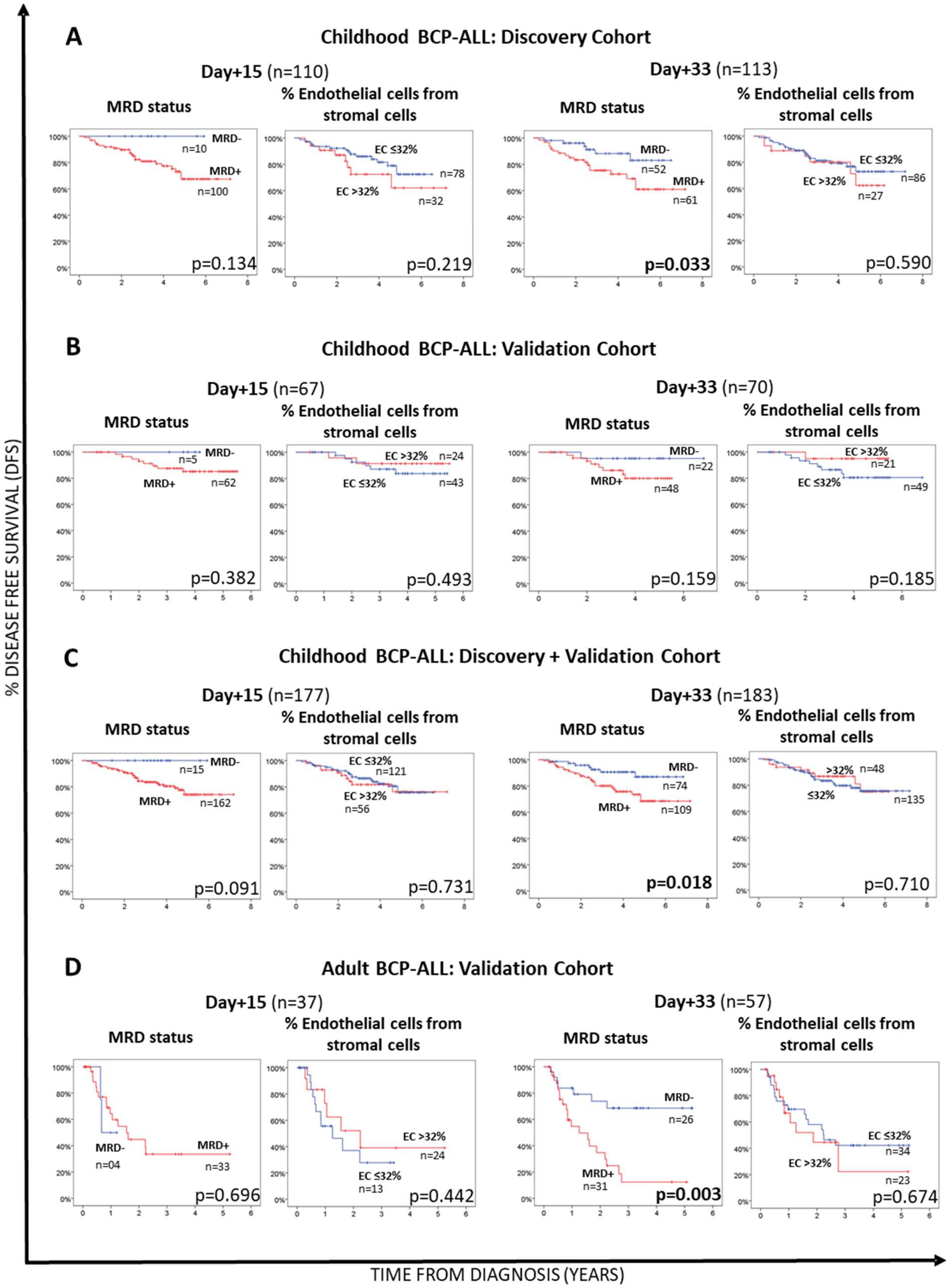

Figure S1. Impact of the BM MRD status and the percentage of BM endothelial cells within the whole BM stromal cell compartment Panels (**A**–**C**) at day +15 and day +33 of therapy on disease free survival (DFS) of childhood BCP-ALL Panel. (**A**) Discovery cohort; (**B**) validation cohort; (**C**) discovery plus validation cohort); adult BCP-ALL Panel (**D**), respectively. Statistical significance was set at $p < 0.05$ (log-rank test).

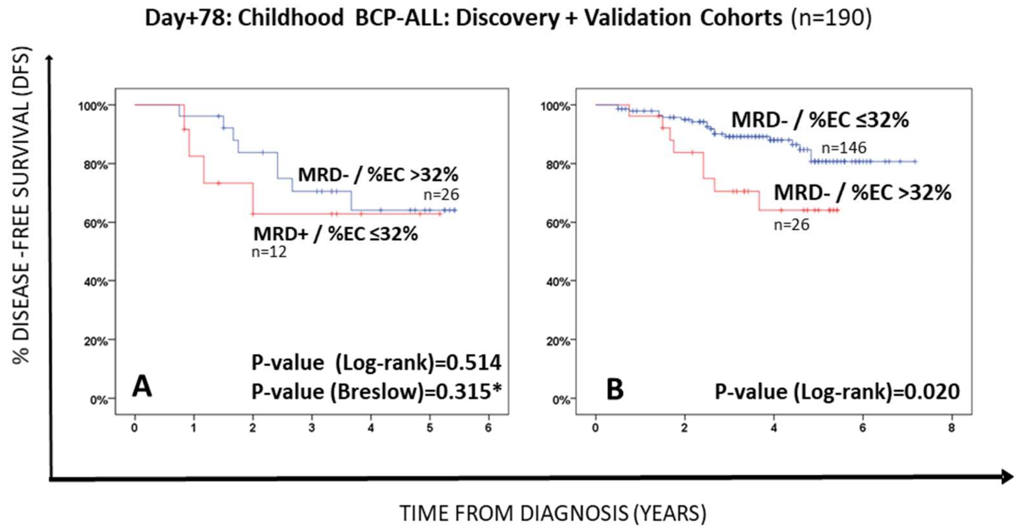

Figure S2. Impact of the bone marrow (BM) minimal residual disease (MRD) status and the percentage of BM endothelial cells (EC) within the whole BM stromal cell compartment at day +78 of therapy on disease-free survival (DFS) of childhood BCP-ALL for both the discovery plus validation cohorts: comparison between MRD$^-$ patients with >32% EC in BM vs. MRD$^+$ cases with ≤32% EC Panel (**A**), and among MRD$^-$ patients between cases with low (≤32%) vs high (>32%) percentages of EC within BM stromal cells Panel (**B**). Statistical significance was set at $p < 0.05$ (log-rank test). * Breslow test was used in Panel A to exclude a possible statically significant difference at the first part of the curve.
